# Carbon Nanofibers Modified Graphite Felt for High Performance Anode in High Substrate Concentration Microbial Fuel Cells

**DOI:** 10.1155/2014/130185

**Published:** 2014-04-22

**Authors:** Youliang Shen, Yan Zhou, Shuiliang Chen, Fangfang Yang, Suqi Zheng, Haoqing Hou

**Affiliations:** ^1^School of Materials Science and Engineering, Nanchang University, Nanchang 330031, China; ^2^Jiangxi Key Laboratory of Surface Engineering, Jiangxi Science & Technology Normal University, Nanchang 330013, China; ^3^Department of Chemistry and Chemical Engineering, Jiangxi Normal University, Nanchang 330022, China

## Abstract

Carbon nanofibers modified graphite fibers (CNFs/GF) composite electrode was prepared for anode in high substrate concentration microbial fuel cells. Electrochemical tests showed that the CNFs/GF anode generated a peak current density of 2.42 mA cm^−2^ at a low acetate concentration of 20 mM, which was 54% higher than that from bare GF. Increase of the acetate concentration to 80 mM, in which the peak current density of the CNFs/GF anode greatly increased and was up to 3.57 mA cm^−2^, was seven times as that of GF anode. Morphology characterization revealed that the biofilms in the CNFs/GF anode were much denser than those in the bare GF. This result revealed that the nanostructure in the anode not only enhanced current generation but also could tolerate high substrate concentration.

## 1. Introduction

Microbial fuel cells (MFCs) are electrochemical devices that use electroactive microorganisms to oxidize organic chemicals and generate electric power [1]. Based on the “green” power source characteristic, the MFCs show great potential in many applications including wastewater treatment, biosensors, water desalination, remote power sources, biohydrogen production, and heavy metal removal and recovery [2–4]. Currently, the limited performance is one of main obstacles for the MFC on the way to practical application.

Anode related to the biofilm growth plays a crucial role on the performance of MFCs. Recently, some measures have been taken to improve the performance of anode, which mainly included architecture design and surface modification. Various macroporous carbons were developed for anodes in MFCs, such as carbon papers [5], carbon cloth [6], graphite rod [7], graphite fiber brush [8], reticulated vitrified carbon (RVC) [7], graphite felt [9], electrospun carbon fiber mats [10], natural plant derived carbon materials [11], and layered corrugated carbon [12]. Simultaneously, some composite materials prepared by surface modification were also studied as high performance anodes in MFCs, such as redox or conducting polymer [13–15] and nanocarbons [16], modified carbon materials [17, 18], and carbon nanotube-coated macroporous polymers [19, 20].

Though the highest anodic current density of 400 A m^−2^ was obtained in one of our previous studies by using layered corrugated carbon [12], the performance of these anodes was measured under relatively low-concentration substrate, for example, below 20 mM acetate. Though a diversity of substrates were employed as substrates in MFCs, including saccharides, alcohols, and different kinds of wastewater, which had been summarized in some review such as [21], the study on the performance of anode in MFCs under high concentration substrate was rare. The tolerance of high concentration substrate would expand the application of MFCs to treat high strength wastewater, thus showing great help for practical application.

In this study, we report carbon nanofiber modified graphite felt (CNFs/GF) for anode in high substrate concentration microbial fuel cells. CNFs/GF anode is prepared by growth of CNFs on GF via chemical vapor deposition. The anodic performance of the CNFs/GF anode in different concentration of acetate is investigated, as well as the behavior of biofilms in the CNFs/GF, and compared with the bare graphite felt.

## 2. Method

### 2.1. Materials Preparation and Characterization

Graphite felt (GF) (Hunan Jiuhua Carbon High-Tech Co., Xiangtan, Hunan, China) was firstly soaked in 10 wt% FeCl_3_ for 1 h and then dried in a vacuum oven at 100°C for 1 h. The growth of carbon nanofibers onto GF was conducted in a furnace equipped with a quartz tube. The GF was heated to 850°C at a rate of 5°C/min in N_2_ atmosphere, then inlet the mixture of H_2_ and N_2_ (H_2_/N_2_ = 1 : 4) at a total flow of 100 mL min^−1^ for 1 h to reduce the Fe (III) to Fe (0). Subsequently, let the furnace cool down to about 750°C and then inlet acetylene with rate of 10 mL min^−1^ for 5 min. After cooling down to room temperature naturally, the CNFs/GF was taken out. The residue Fe in the CNFs/GF was removed by socking it in 0.5 M hydrochloric acid solution and rinsed with distilled water. At last, the samples were dried in the drying oven at 100°C for 1 h. The morphology characterization of samples was observed by a Tescan Vega-3 scanning electron microscope (SEM).

### 2.2. Electrode Preparation

Graphite plate (GP) cut into pieces with size of 1 × 1 cm^2^ was connected with stainless wire and encapsulated by epoxy resin. One side of GP was polished by 2000 mesh sandpaper and used as support for anode electrode. The CNFs/GF and GF were cut into pieces with the same size as the GP and glued onto the polished GP by conductive glue.

### 2.3. Electrochemical Measurement

Primary domestic wastewater was collected from the wastewater treatment plant (Qingshan, Nanchang, China) and used as the inoculum to select secondary biofilms through procedures following previous report [11]. All current density data in this paper refer to secondary biofilms and the electrochemical performance tests were conducted when the biofilms activity reached stationary level.

The electrochemical measurements were carried out in three-electrode half-cell, in which a 500 mL bottle was assembled with six working electrodes, one Ag/AgCl reference electrode (saturated KCl, 0.198 V versus standard hydrogen electrode (SHE)) and one carbon felt counter electrode (8 cm^2^). The experiments were carried out with computer controlled potentiostat (CHI1040B) which was equipped with eight channels in parallel. For the chronoamperometric (CA) measurement, a potential of +0.2 V was applied onto the working electrodes and the current was recorded. All experimental operations were conducted anaerobically at 35°C which was the optimal growth temperature of bacteriain 50 mM phosphate buffer solution (pH = 7.0) with different concentrations of acetate substrate. All of the electrode potentials were given as versus Ag/AgCl and all of the current density values were normalized to the projected surface area.

### 2.4. Biofilm SEM Imaging

The morphology of the biofilm was characterized by scanning electron microscopy (SEM). The biofilm samples for SEM characterization were prepared as follows [11], biofilm samples were firstly fixed by 5 wt% glutaric aldehydes in 50 mM phosphate buffer solution (pH = 7.0), then dehydrated in a graded series of ethanol aqueous solution (10%, 25%, 40%, 55%, 70%, 80%, 90%, and 100%), and finally dried naturally at room temperature. After coating a layer of gold, the biofilm samples were observed under SEM.

## 3. Results and Discussion

### 3.1. Morphologies of GF and CNFs/GF

**Figure 1:**
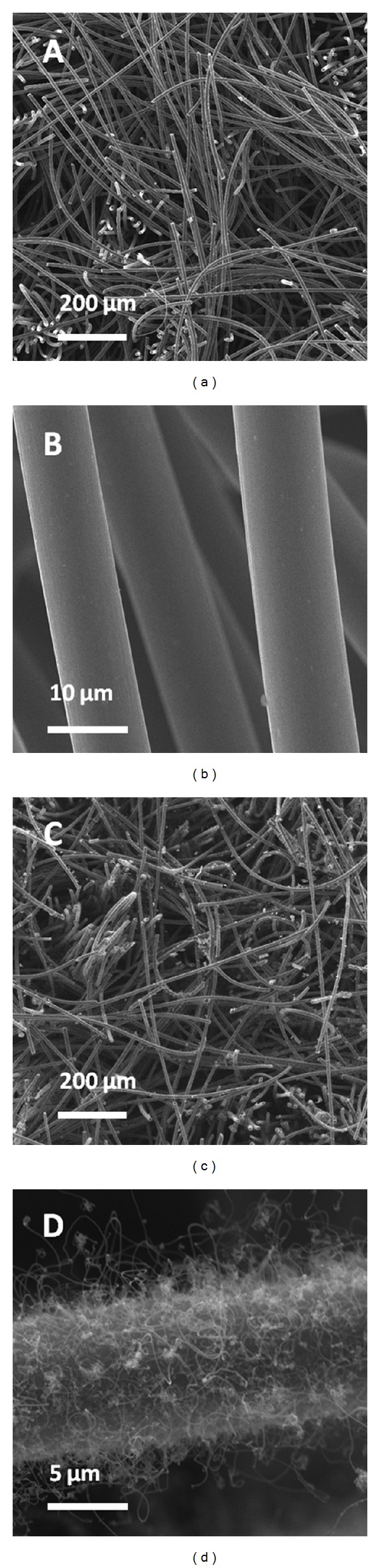
SEM images of ((a) and (b)) GF and ((c) and (d)) CNFs/GF.

Figure 1 shows the SEM images of GF and CNFs. The diameter of graphite fiber in the GF is about 10 *μ*m. The GF has a macroporous structure with pore size in the tens of micrometers. Detailed SEM image in Figure 1(b) shows that the surface of GF is smooth. After a CVD process, a layer of long length carbon nanofibers with diameter of about 100 nm was successfully grown onto the graphite fibers surface to form CNFs/GF composite (Figure 1(d)), which is in accordance with the micro/nano structures of carbon composite in [22]. The CNFs/GF displays a hierarchical micro-/nanostructures which would be beneficial for the attachment of bacteria to the anode and enhancement of electron transfer from inside bacteria to the anode simultaneously.

### 3.2. Biocatalytic Current Generation of GF and CNFs/GF Anode

**Figure 2:**
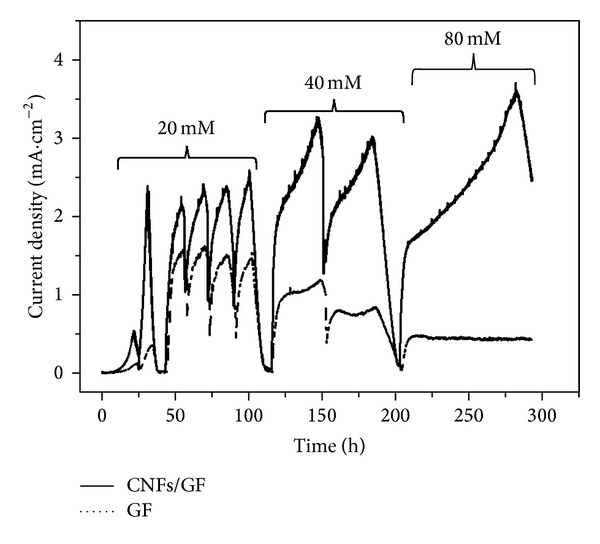
Biocatalytic current generation curves of GF (curves of dotted line) and CNFs/GF (curves of solid line) under different concentration of acetate measured in a half cell at 35°C. The arrows indicate the replenishment of the substrate with different concentration of acetate.

The biocatalytic current generation curves of GF and CNFs/GF anodes under different concentration of acetate are shown in Figure 2. It can be observed that the CNFs/GF anode achieves the maximum current density at the second cycle, while the GF requires three cycles. This result demonstrates the CNFs modification enhances the attachment of the bacteria to the anode. After running for six cycles under low acetate concentration of 20 mM, the GF and CNFs/GF anodes all delivered a stable maximum current density. The CNFs/GF anode generates a maximum geometric current density of about 2.42 mA cm^−2^, which is 54% higher than that from GF of about 1.57 mA cm^−2^. By increasing the concentration of acetate to 40 mM, the current density of GF anode shows a slight decrease to 1.17 mA cm^−2^, while the CNFs/GF anode produces a much higher current density of 3.23 mA cm^−2^, about 33% higher than that under 20 mM acetate. By further increasing the concentration of acetate to 80 mM, the maximum current density of the CNFs/GF anode keeps increasing to about 3.57 mA cm^−2^, in contrast, that of the bare GF anode greatly decreases to only about 0.50 mA cm^−2^. It could be concluded that the CNFs/GF anode shows excellent performance of tolerating high strength substrate and that excellent performance is brought by the CNFs modification. It was reported that nanocarbons modification could enhance the attachment of the biofilm and the electron transfer from inside bacteria to the surface of anode and thus increased and stabled the anodic current generation [16–18, 22]. In this paper, the CNFs modification not only increases the current generation, but also shows high performance of tolerating high strength substrate. That could also be attributed to the enhancement of electron transfer (respiration) by the CNFs modification and acceleration of the metabolism of the biofilms in the anode.

### 3.3. Morphology of Biofilms on GF and CNFs/GF

**Figure 3:**
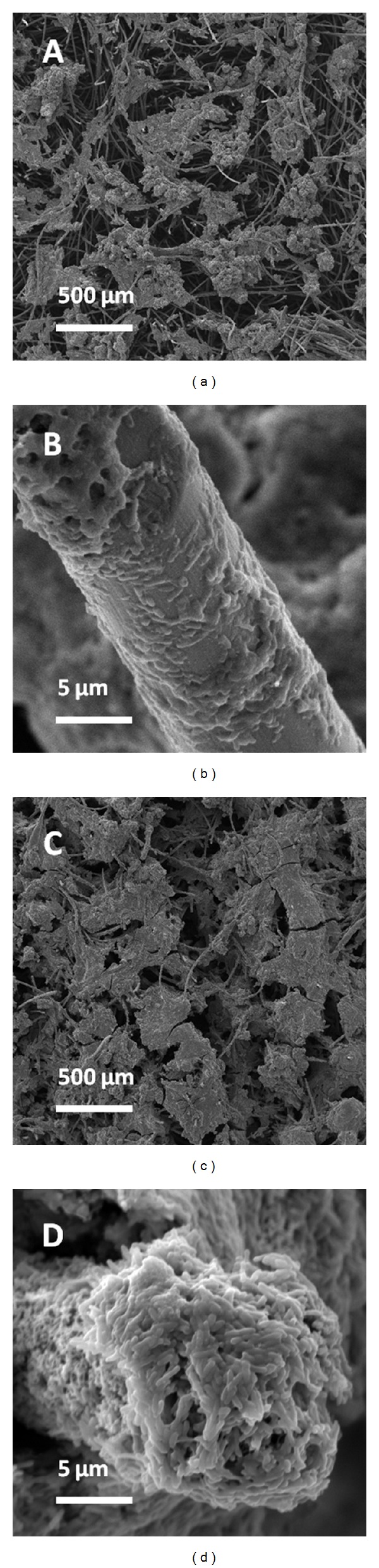
Overview and detailed SEM images of ((a) and (b)) biofilms in GF and ((c) and (d)) CNFs/GF.

In order to verify the anodic performance, after current generation in media with 80 mM acetate, the GF and CNFs/GF anodes with biofilms are taken out. After fixation and drying, the biofilms in the GF and CNFs/GF are observed under the SEM.  Figure 3 shows the images of biofilms grown in the GF and CNFs/GF. It can be seen from the overview images (Figures 3(a) and 3(c)) that the biofilms grown in the CNFs/GF anode are denser than those in the GF anode. Detailed observation of biofilms on individual fiber (Figures 3(b) and 3(d)) reveals that the biofilms on bare graphite fibers are sparse, while those on the CNFs modified fibers are very thick and have a thickness of about 5 *μ*m on individual graphite fiber according to the SEM image in Figure 3(d). That confirms that the current generation for the CNFs/GF is higher than that of the bare GF.

## 4. Conclusion

The performance of tolerating high strength substrate for the CNFs modified GF was demonstrated. The CNFs/GF not only generate enhanced current density in low strength acetate media of 20 mM comparing to the bare GF but also could generate a much higher current density of 3.57 mA cm^−2^ in high strength acetate media of 80 mM, which was 7 times higher than that generated from bare GF. The performance of tolerating high strength substrate was attributed to the nanostructured CNFs which enhanced the electron transfer from inside bacteria to anode (respiration) and accelerated the metabolism of bacteria. Anode materials tolerating high strength substrate would expand the applications of MFCs in high concentration of substrate environment, for example, high strength wastewater treatment from oil and food industry.
